# Robot-Assisted End-Effector-Based Stair Climbing for Cardiopulmonary Exercise Testing: Feasibility, Reliability, and Repeatability

**DOI:** 10.1371/journal.pone.0148932

**Published:** 2016-02-05

**Authors:** Oliver Stoller, Matthias Schindelholz, Kenneth J. Hunt

**Affiliations:** Institute for Rehabilitation and Performance Technology, Division of Mechanical Engineering, Department of Engineering and Information Technology, Bern University of Applied Sciences, Burgdorf, Switzerland; Politehnica University of Bucharest, ROMANIA

## Abstract

**Background:**

Neurological impairments can limit the implementation of conventional cardiopulmonary exercise testing (CPET) and cardiovascular training strategies. A promising approach to provoke cardiovascular stress while facilitating task-specific exercise in people with disabilities is feedback-controlled robot-assisted end-effector-based stair climbing (RASC). The aim of this study was to evaluate the feasibility, reliability, and repeatability of augmented RASC-based CPET in able-bodied subjects, with a view towards future research and applications in neurologically impaired populations.

**Methods:**

Twenty able-bodied subjects performed a familiarisation session and 2 consecutive incremental CPETs using augmented RASC. Outcome measures focussed on standard cardiopulmonary performance parameters and on accuracy of work rate tracking (RMSE_P_−root mean square error). Criteria for feasibility were cardiopulmonary responsiveness and technical implementation. Relative and absolute test-retest reliability were assessed by intraclass correlation coefficients (ICC), standard error of the measurement (SEM), and minimal detectable change (MDC). Mean differences, limits of agreement, and coefficients of variation (CoV) were estimated to assess repeatability.

**Results:**

All criteria for feasibility were achieved. Mean V′O_2peak_ was 106±9% of predicted V′O_2max_ and mean HR_peak_ was 99±3% of predicted HR_max_. 95% of the subjects achieved at least 1 criterion for V′O_2max_, and the detection of the sub-maximal ventilatory thresholds was successful (ventilatory anaerobic threshold 100%, respiratory compensation point 90% of the subjects). Excellent reliability was found for peak cardiopulmonary outcome measures (ICC ≥ 0.890, SEM ≤ 0.60%, MDC ≤ 1.67%). Repeatability for the primary outcomes was good (CoV ≤ 0.12).

**Conclusions:**

RASC-based CPET with feedback-guided exercise intensity demonstrated comparable or higher peak cardiopulmonary performance variables relative to predicted values, achieved the criteria for V′O_2max_, and allowed determination of sub-maximal ventilatory thresholds. The reliability and repeatability were found to be high. There is potential for augmented RASC to be used for exercise testing and prescription in populations with neurological impairments who would benefit from repetitive task-specific training.

## Introduction

Substantial efforts are being made globally to promote regular cardiovascular exercise to positively modify risk factor profiles in apparently healthy individuals as well as in various disease populations [1]. Physical activities that demand a strong increase in cardiovascular stress by including dynamic exercise of large muscle groups is recommended, with appropriate dosage of 3 to 6 times per week for a minimum of 30 minutes per session at a minimum intensity of 40% to 60% of the maximal exercise capacity (V′O_2_max) [2]. Thus, the assessment of cardiovascular fitness is important for exercise prescription and evaluation in clinical practice and for research purposes to evaluate the effects of exercise interventions.

Cardiopulmonary exercise testing (CPET) is considered the ‘gold standard’ for assessment of cardiovascular fitness and has proven to be an important diagnostic tool in healthy individuals and those with various medical conditions [3, 4]. The most common modes for CPET are treadmill exercise and leg cycle ergometry. While walking on the treadmill requires superior motor skills and is therefore not the primary choice in disabled populations, leg cycle ergometry is well established among individuals with physical limitations. There are considerable limitations to implementation of CPET in populations with severe motor limitations caused by stroke, multiple sclerosis, spinal cord injury, traumatic brain injury, or cerebral paresis [5, 6]. Although alternative approaches such as semi-recumbent cycle ergometry [7] or total-body recumbent stepping [8] have been proposed, repetitive task-specific testing and training environments for individuals with disabilities are lacking. Considering that walking ability, including stair climbing, are important factors for independence and therefore major goals during neurological rehabilitation, and given the importance of repetitive task-specific training to facilitate motor recovery [9, 10], advanced CPET and intervention strategies which incorporate walking and stair climbing modalities are of importance to facilitate the assessment and improvement of cardiovascular fitness in populations with severe neurological impairments.

A promising approach to overcome motor limitations while facilitating task-specific activity and cardiovascular stress is robot-assisted end-effector-based stair climbing (RASC) [11]. Individuals stand on footplates whose trajectories simulate the stance and swing phases of normal gait and stair ascent/descent. The technology provides important features for neuromuscular and cardiovascular training and the opportunity to implement control strategies to increase participation and effort. Initial studies have shown that cardiopulmonary responses during the simulation of walking were similar to floor walking [12, 13]. However, RASC did not provoke cardiovascular stress comparable to conventional stair climbing, and active contribution was noted to be essential to achieve high cardiovascular stress within certain desirable ranges of predicted V′O_2_max [13]. Therefore, as described in the sequel, RASC has been augmented with a visual feedback-control system to guide exercise intensity. A novel algorithm estimates the exercise work rate, which allows the implementation of standardised CPET protocols and training environments.

As a first step towards verifying the system for future application in neurologically impaired populations, the aim of this study was to evaluate the feasibility, reliability, and repeatability of augmented RASC-based CPET in able-bodied subjects. The driving questions were: (1) does the approach provide substantial cardiopulmonary responses appropriate for CPET; (2) is the feedback-control approach technically implementable; and (3) is the approach able to reliably determine V′O_2_max, sub-maximal ventilatory thresholds, and other key outcomes?

## Materials and Methods

### Subjects

Twenty able-bodied subjects (11 female; Table 1) were recruited between December 2014 and April 2015. Eligibility criteria were: (1) age 18–50 years, (2) physically healthy, (3) no cardiovascular, pulmonary or musculoskeletal problems that may interfere with or contraindicate CPET, and (4) a positive Physical Activity Readiness Questionnaire [14].

**Table 1:** Gender specific characteristics and primary outcome data^a^.

	Female (n = 11)	Male (n = 9)
Age [years]	29.15±6.14	27.45±2.26
Body mass [kg]	62.91±11.60	82.89±10.42
Height [m]	1.69±0.07	1.83±0.09
BMI [kg/m^2^]	21.97±3.06	24.65±1.58
Resting HR [beats/min]	68.91±9.74	62.67±5.63
Predicted HR_max_ [beats/min]	187.60±4.30	188.79±1.58
Achieved HR_peak_ [beats/min]	185.68±7.55	187.33±7.05
Achieved % of predicted HR_max_ [%]	98.98±3.28	99.22±3.53
Predicted V′O_2max_ [L/min]	2.40±0.38	3.95±0.52
Achieved V′O_2peak_ [L/min]	2.64±0.51	4.01±0.93
Achieved % of predicted V′O_2max_ [%]	109.70±8.63	102.34±8.82
Predicted P_max_ [W]	152.65±26.58	226.57±40.24
Achieved P_peak_ [W]	128.93±33.38	195.41±26.15
Achieved % of predicted P_max_ [%]	83.72±11.88	87.15±8.33
VAT [L/min]	1.59±0.34	2.23±0.31
VAT [% of V′O_2peak_]	60.48±7.17	55.42±4.41
RCP [L/min]	^b^ 2.28±0.49	3.45±0.43
RCP [% of V′O_2peak_]	^b^ 86.63±6.62	86.02±5.82

BMI, Body mass index; HR, Heart rate; V′O_2_, Oxygen uptake; P, Work rate (power); VAT, Ventilatory anaerobic threshold; RCP, Respiratory compensation point

^a^ Values are given in numbers (n) or mean ± standard deviation of the repeated trials

^b^ n = 10

All subjects were informed about risks and benefits, and gave signed informed consent prior to participation. The Ethics Review Board of the Canton of Bern in Switzerland approved the study (reference No. 155/12).

### Technical implementation

**Fig 1:**
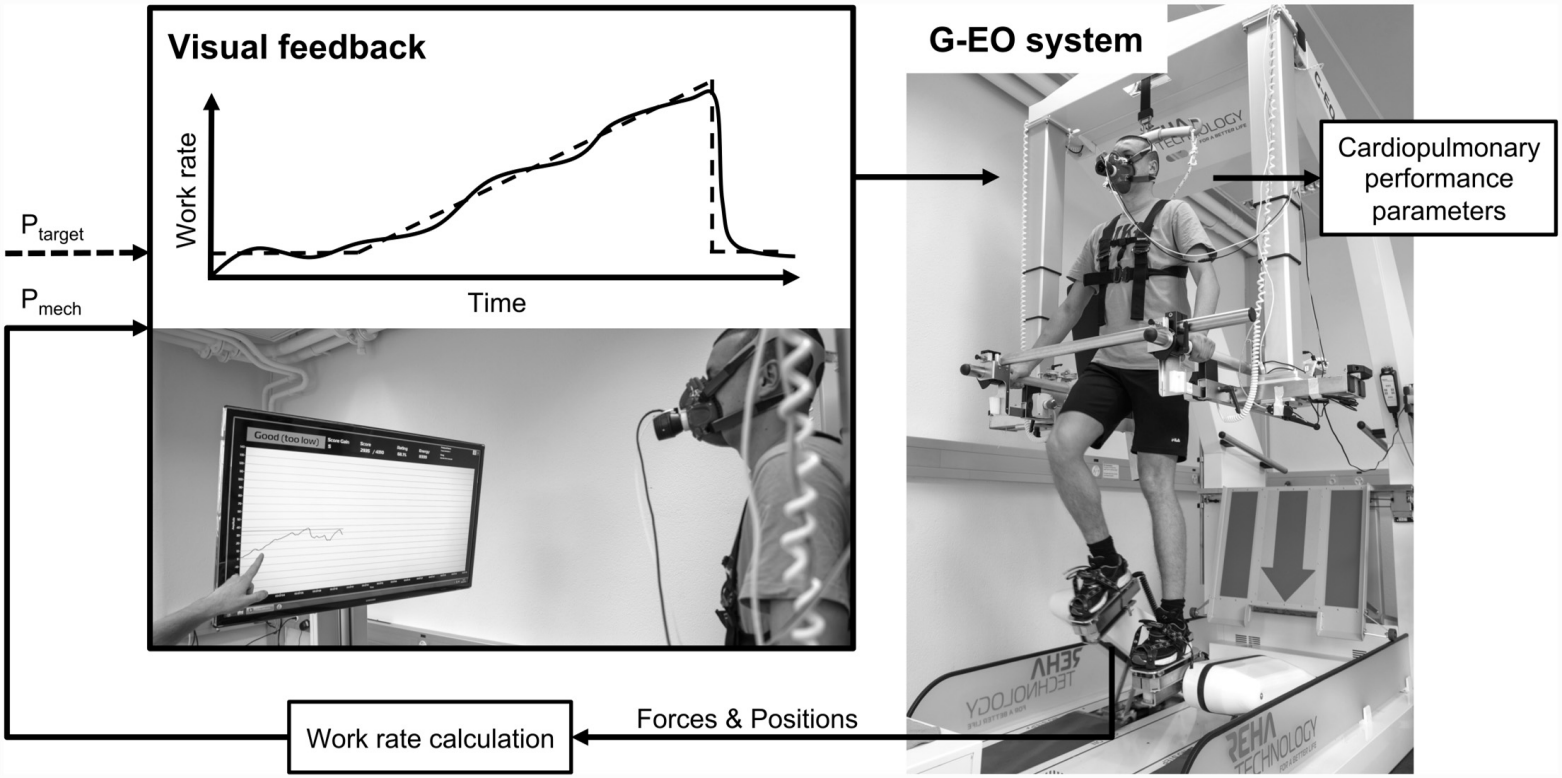
Augmented robot-assisted end-effector-based stair climbing (RASC) using the G-EO system. Forces and positions are measured in real time to allow calculation of the mechanical work rate (P_mech_, solid line) and projection onto a screen in front of the person. Individual target work rate profiles (P_target_, dashed line) are used to guide exercise intensity. Physiological variables are monitored continuously.

An end-effector-based robotic device (G-EO System Evolution, Reha Technology AG, Switzerland) was used to implement stair climbing in a standard setting (step height 18 cm, cadence 70 steps/min) (Fig 1). The subject’s feet were fixed on footplates, which were equipped with 4 rectangularly arranged force sensors. The positions of the footplates were captured via a modified programmable logic controller interface. An algorithm was developed to calculate the subject’s mechanical work rate (P_mech_) during robotic end-effector-based stair climbing based on force and velocity data (derivates of positions). Briefly, active loading of the leg during the stance phase and active pulling during the swing phase were counted as positive power, whereas any assistance of the robot (that resulted in passivity of the subject) was counted as negative power. A human-in-the-loop feedback system was implemented to allow the subjects to volitionally modify their effort to meet the work rate target (Fig 1). P_mech_ was sampled at 20 Hz and filtered by a first order infinite impulse response (IIR) low pass filter with Butterworth topology (cut off frequency 0.03 Hz), and projected onto a monitor in front of the device for the subject to see, together with a target mechanical work rate (P_target_). The subjects were instructed to follow P_target_ by actively adapting their stair climbing effort.

### Experimental protocol

All subjects performed a familiarisation session and 2 incremental exercise tests (IET) to volitional exhaustion (trial 1 and trial 2). The familiarisation session started with detailed information on the test procedures, safety procedures and potential adverse events. Subjects were then positioned within the device and measurement sensors were fitted (respiratory mask, fixation of the feet, chest harness, handrail height, heart rate belt), and a standardised stair climbing protocol was implemented to familiarise the subjects with the feedback-control system. Thereby, subjects had to follow a predefined P_target_ profile for 15 min, which consisted of 60 alternating P_target_ levels (15 seconds per step, range: -10 W– 70W). Criterion for a successful familiarisation was a real-time work rate tracking score of >60%, calculated by a per-second rating of 0–5 W deviation = 100% accuracy, 5–10 W deviation = 50% accuracy, >10 W deviation = 0% accuracy. The familiarisation session was extended in 5 min steps until the subject reached the predefined score.

Following a resting period of 5 min, the maximal voluntary effort within the system was evaluated to predict the maximal work rate (P_max_) for the subsequent tests. Subjects had to follow a steep slope (P_target_ slope of 200 W/min) until they reach their power limit. The maximal P_mech_ value achieved was defined as P_max_ for the following 2 trials.

After a break of at least 24 h, subjects then performed 2 IETs on separate days with 48–72 h rest between the trials. All sessions were controlled for time of day. Subjects were instructed to avoid additional strenuous activity during participation in the study and not to consume food, alcohol, nicotine or caffeine at least 3 h prior to testing.

Both IETs started with a 10 min warm-up at 20% of P_max_. Subjects then had a 2 min break to drink water and to adjust the respiratory mask before the 4-phase IET protocol started: (1) standing (rest)—subjects stood on the footplates for 5 min, (2) ‘passive’ stair climbing—subjects climbed stairs without visual feedback, i.e. there was no control of work rate which means that subjects relied on robotic assistance to lift up their body mass during stair climbing, (3) ‘active’ stair climbing—subjects actively climbed by following P_target_ on the screen, i.e. the robotic assistance was neutralised by the feedback-control approach which means that subjects had to actively raise their body mass and could further increase P_mech_ by volitional pulling and pushing on the footplates within the gait trajectory, (4) recovery—subjects climbed stairs passively without visual feedback as described above. The progressive ramp (active stair climbing phase) was defined as a continuous slope aiming to reach the predefined P_max_ in 10 min. Termination criteria were: (1) volitional exhaustion, (2) P_mech_ below P_target_ for 60 sec, or (3) specific symptoms such as severe or unusual shortness of breath, signs of poor perfusion, chest pain, etc., according to established CPET guidelines from the American College of Sports Medicine [3].

### Outcome measures

Cardiopulmonary performance parameters were recorded by a breath-by-breath cardiorespiratory monitoring system (MetaMax 3B, Cortex Biophysik, Germany). Calibration was done prior to each test using a 3 L syringe, atmospheric pressure (mmHg), and both ambient and precision-reference gases (5.00% CO_2_, 15.00% O_2_, rest N_2_). Heart rate was recorded by a heart rate belt (T31, Polar Electro, Finland) and a receiver board (HRMI, Sparkfun, USA).

Primary outcome measures were: peak oxygen uptake (V′O_2peak_), peak heart rate (HR_peak_), peak power (P_peak_), the first ventilatory threshold (ventilatory anaerobic threshold, VAT), and the second ventilatory threshold (respiratory compensation point, RCP). These designations of the sub-maximal ventilatory thresholds follow the recommendations of Binder et al. [15]. Secondary outcome measures were: time to V′O_2peak_ (tV′O_2peak_), peak ventilation rate (V′_Epeak_), peak respiratory rate (R_fpeak_), respiratory exchange ratio (RER) at V′O_2peak_ (RER_peak_), oxygen cost of work (ΔV′O_2_/ ΔP_mech_), ventilatory efficiency (ΔV′_E_/ΔV′CO_2_), accuracy of work rate tracking (RMSE_P_), oxygen cost of passive stair climbing (ΔV′O_2rest-passive_), and heart rate response of passive stair climbing (ΔHR_rest-passive_).

The criteria for feasibility assessment were: (i) cardiopulmonary responsiveness (is the approach able to provoke substantial cardiovascular responses and to determine V′O_2max_ and ventilatory thresholds?) and (ii) technical implementation (are the subjects able to follow P_target_ during CPET, i.e. successful feedback-control approach?). The concept was considered to have satisfied cardiopulmonary responsiveness if: (1) V′O_2peak_ and HR_peak_ values were comparable to or significantly higher than predicted V′O_2max_/HR_max_, (2) ≥95% of the subjects achieve 1 criterion of V′O_2max_, and (3) at least 1 sub-maximal ventilatory threshold (VAT/RCP) can be detected. The technical implementation was considered satisfied by a mean work rate tracking error (RMSE_P_) of ≤ 5 W during the active stair climbing phase (excluding the last 60 seconds of the incremental slope).

### Data processing

Raw breath-by-breath data were averaged over 15 breaths [16]. Peak cardiopulmonary variables were determined as the maximal values during incremental exercise. P_peak_ was defined as the highest P_mech_ value achieved. Predicted V′O_2max_ was estimated from a model including gender, age, body mass index, resting heart rate, and self-reported physical activity [17]. Predicted HR_max_ was defined as 208 − (0.7 × age) [18]. Criteria for the achievement of V′O_2max_ were: (1) plateau in oxygen uptake, (2) HR_peak_ ≥ predicted HR_max_, and (3) RER_peak_ ≥ 1.15 [3, 19]. The identification of a plateau in oxygen uptake was performed by plotting the slope and 95% confidence interval (CI) of the V′O_2_-P_mech_ slope, where data points outside the extrapolated 95% CI were taken as evidence of plateauing or levelling-off behaviour [20].

Two experienced independent raters estimated the VAT and the RCP visually based on the criteria detailed by Binder et al. [15]. The VAT was determined by the combination of these criteria: (1) the minimum point of the ventilatory equivalent of oxygen (V′_E_/V′O_2_), or the point of its first increase without a simultaneous increase in the ventilator equivalent for carbon dioxide (V′_E_/V′CO_2_); (2) the minimum point of the partial pressure of end-tidal oxygen tension (P_ET_O_2_), or the point of its first increase, without a decrease in the partial pressure of the end-tidal CO_2_ tension (P_ET_CO_2_); and (3) the deflection point of V′CO_2_ vs. V′O_2_ (‘V-slope method’). The RCP was determined by inspection of: (1) the minimum point or non-linear increase of V′_E_/V′CO_2_, (2) the turning point of P_ET_CO_2_, and (3) the deflection point of V′_E_ vs. V′CO_2_. The agreement between the raters (interrater reliability) was excellent (intraclass correlation coefficient (ICC_2,1_): VAT 0.943 (CI95% 0.890–0.970), RCP 0.976 (CI95% 0.951–0.988)). Disagreements were resolved by discussion between the two raters.

The accuracy of work rate tracking was expressed by the root mean square error (RMSE_P_) between P_target_ and P_mech_. Steady-state during standing and passive walking was defined by excluding the first 2 minutes and last minute of each phase, i.e. steady-state calculations were done using data from the 3^rd^– 4^th^ minute of a given phase. Oxygen cost and heart rate response of passive stair climbing was defined as the difference between standing and passive stair climbing steady-state values. Data processing was performed using MATLAB (Version R2012a, MathWorks, USA), LabVIEW (Version 2011, National Instruments, USA), and MetaSoft 3 (Version 3.9.9 SR5, Cortex Biophysik, Germany).

### Statistical analysis

All data were tested for normality. Paired-sample T-tests were applied to compare mean differences and potential practice effects. The data of the repeated trials were averaged for comparison with predicted values. Reliability was quantified using intraclass correlation coefficients (ICC_2,1_) with 95% CI [21]. ICC results of 0.60–0.74 were considered as ‘good’, and ICC results >0.74 as ‘excellent’ [22]. Absolute reliability was determined by estimating the standard error of measurement (SEM = standard deviation of the difference (SD_diff_)$\sqrt{1-ICC}$) and the minimal detectable change (MDC = 1.96 x $\sqrt{2}$ x SEM), presented in absolute values and percentages. Repeatability was estimated by mean difference (MD), limits of agreement (LoA) (MD ± 1.96 x SD_diff_), and coefficients of variation (CoV) (SD_diff_/mean). Two-sided p-values p ≤ 0.05 were considered significant. Statistical analyses were performed using MATLAB (Version R2012a, MathWorks, USA) and SPSS (Version 22.0, IBM, USA).

## Results

**Fig 2:**
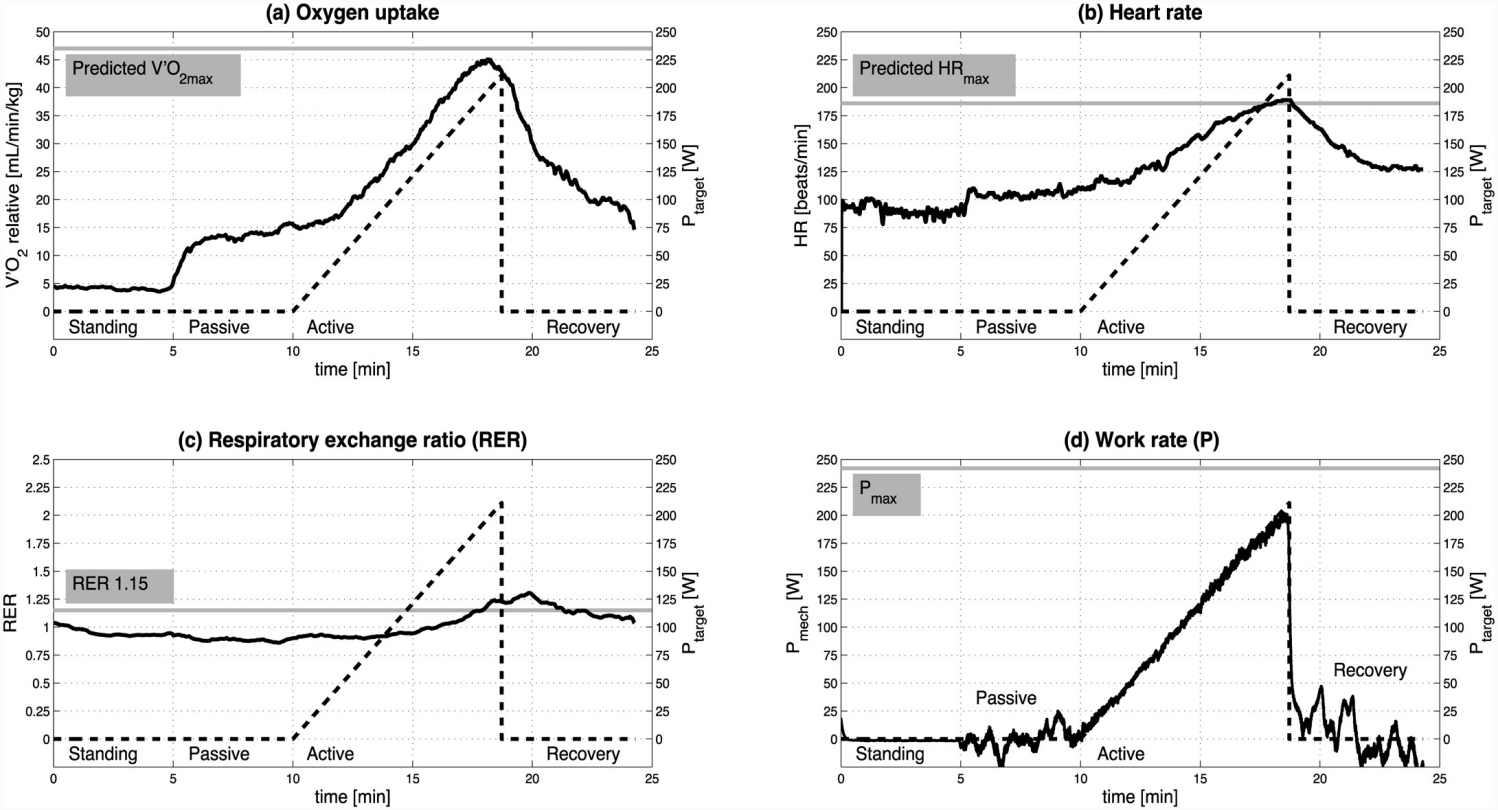
Representative experimental data of RASC-based CPET. Data represent a male subject, trial 1. (a) Oxygen uptake and predicted V′O_2max_ [17], (b) heart rate and predicted HR_max_ [18], (c) Respiratory exchange ratio (RER) and RER threshold of 1.15, (d) calculated mechanical work rate (P_mech_), target work rate (P_target_) and predicted maximal work rate (P_max_, evaluated during familiarisation).

All subjects were able to achieve a real-time work rate tracking score of >60% within the first 15 min of practicing with the feedback-control approach during the familiarisation session. No adverse events occurred during the CPET sessions; all subjects performed both trials without complications. The main reason for test termination was inability to reach P_target_ due to generalized and/or leg fatigue. Representative experimental data of RASC-based CPET in a male subject (trial 1) illustrate the method (Fig 2).

**Fig 3:**
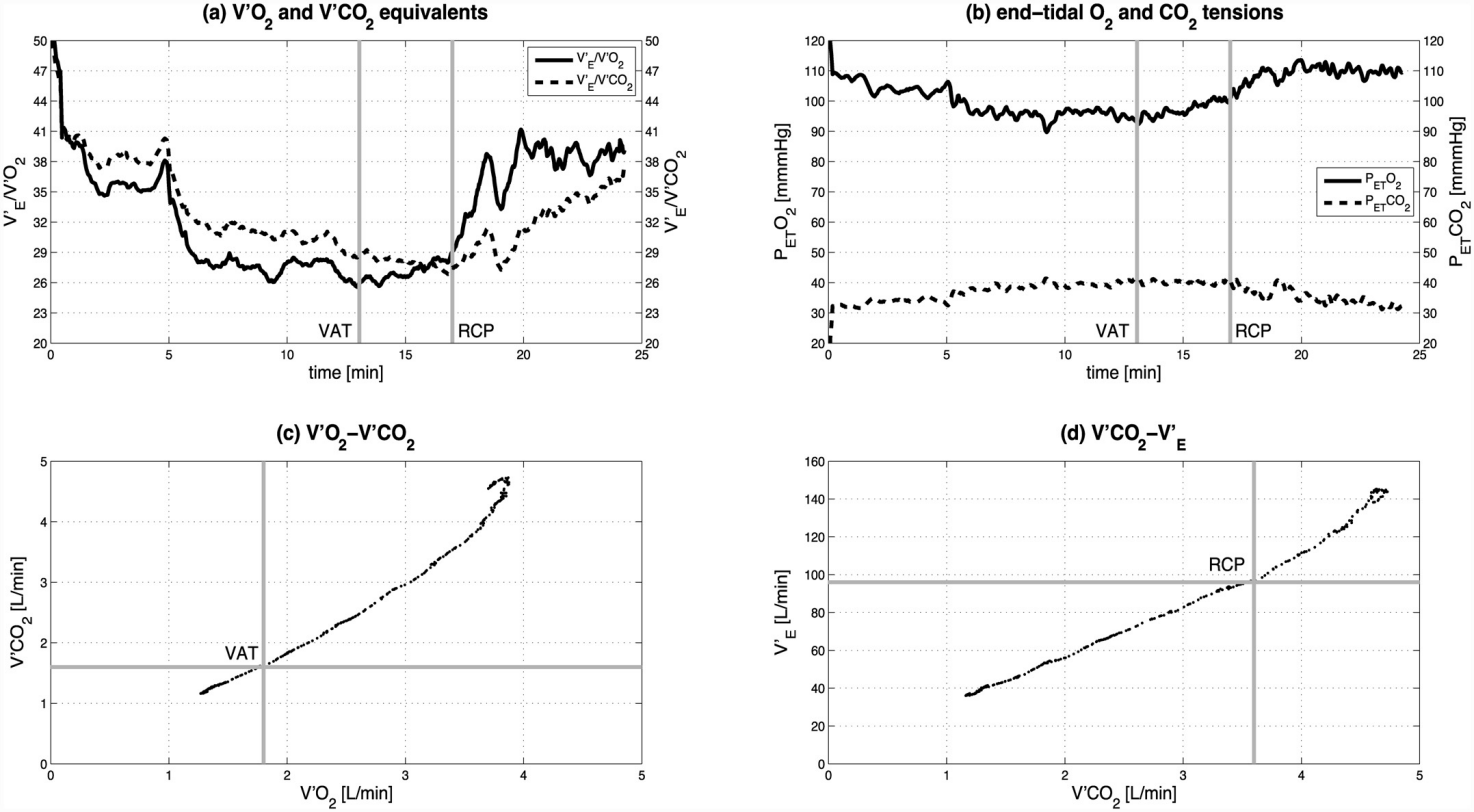
Determination of the ventilatory anaerobic threshold (VAT) and the respiratory compensatory point (RCP). Data represent a male subject, trial 1. (a) VAT is at the minimum point of V′_E_/V′O_2_ and RCP at the minimum of V′_E_/V′CO_2_, (b) VAT is at the minimum point of P_ET_O_2_ and RCP at the deflection point of P_ET_CO_2_, (c) VAT is at the deflection point of V′CO_2_ vs. V′O_2_ (‘V-slope method’), (d) RCP is at the deflection point of V′_E_ vs. V′CO_2_.

**Fig 4:**
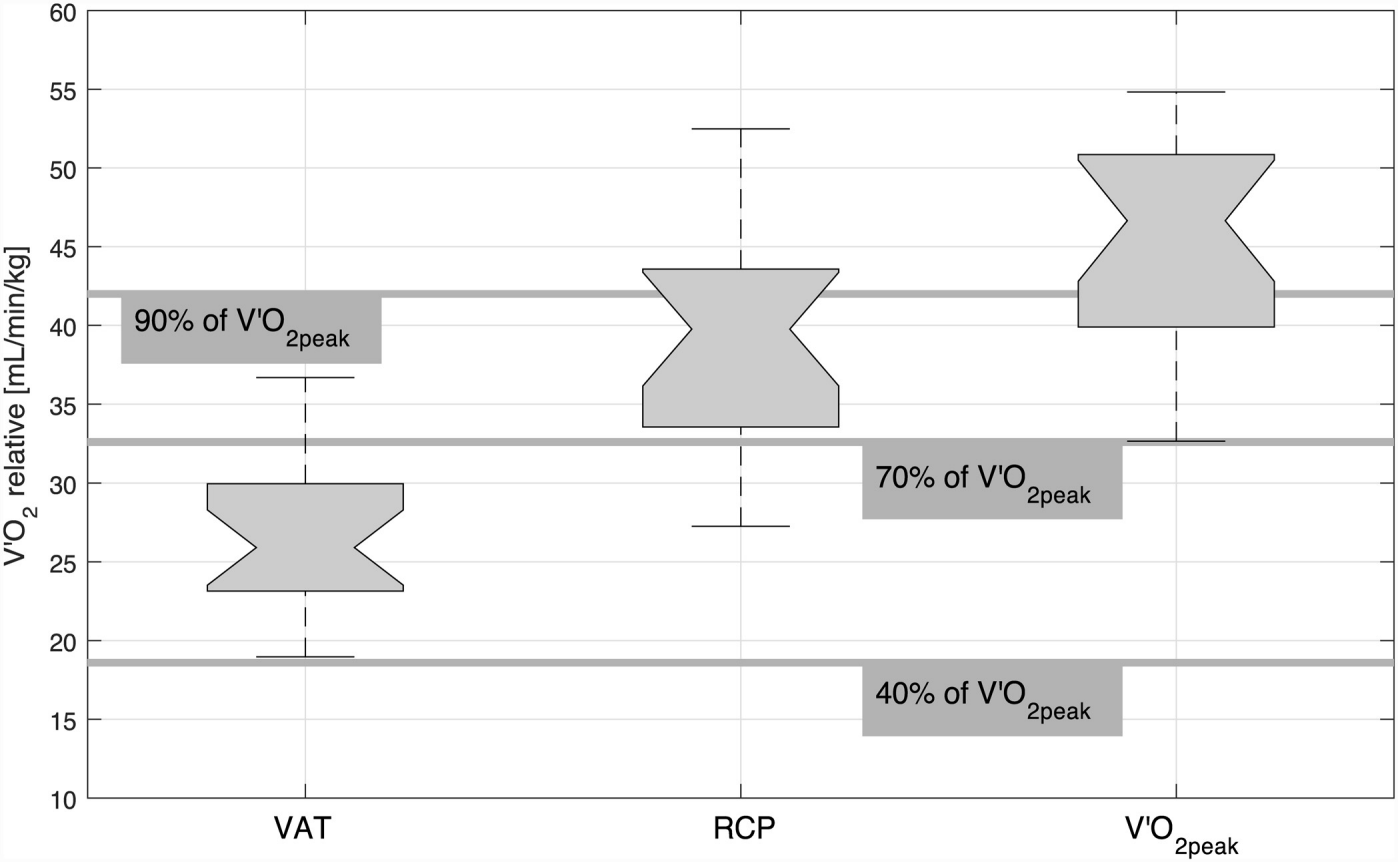
Mean ventilatory anaerobic threshold (VAT), respiratory compensatory point (RCP), and peak oxygen uptake (V′O_2peak_) of the repeated trials. Boxplots show the median (with interval endpoints), the 25^th^ and 75^th^ percentiles (interquartile range, IQR) and the 99.3% coverage (1.5 x IQR). The 40%, 70%, and 90% ranges of V′O_2peak_ are given for orientation.

All criteria for feasibility (cardiopulmonary responsiveness and technical implementation) were achieved. Mean V′O_2peak_ was significantly higher compared to predicted V′O_2max_ in females (2.64±0.51 L/min vs. 2.40±0.38 L/min [mean ± standard deviation], p = 0.004) and comparable to predicted V′O_2max_ in males (4.01±0.93 L/min vs. 3.95±0.52 L/min, p = 0.598) (Table 1). There was no difference between measured mean HR_peak_ and predicted HR_max_ (females: 185.68±7.55 beats/min vs. 187.60±4.30 beats/min, p = 0.330; males: 187.33±7.05 beats/min vs. 188.79±1.58 beats/min, p = 0.518). P_peak_ was significantly lower than predicted P_max_ (females: 128.93±33.38 W vs. 152.65±26.58 W, p<0.001; males: 195.41±26.15 W vs. 226.57±40.24 W, p = 0.004). 95% of the subjects achieved 1 criterion, 50% of the subjects achieved 2 criteria, and 25% of the subjects achieved all criteria for V′O_2max_ (Table 2). RER ≥ 1.15 was the most frequent criterion achieved (75%; plateau in oxygen uptake: 40%; HR_peak_ ≥ HR_max_: 25%). The VAT was detected in all subjects and in both trials, and an RCP was found in 95% of the subjects and 90% of the trials (detection not possible in both trials of 1 female subject). The method to determine the VAT and the RCP is illustrated in Fig 3 (male subject, trial 1). V′O_2_ at VAT was 1.59±0.34 L/min (60.48% of V′O_2peak_) for females and 2.23±0.31 L/min (55.42% of V′O_2peak_) for males. V′O_2_ at RCP was 2.28±0.49 L/min (86.63% of V′O_2peak_) for females and 3.45±0.43 L/min (86.02% of V′O_2peak_) for males. The mean VAT, RCP and V′O_2peak_ values of the 2 trials are illustrated in Fig 4. All subjects achieved an RMSE_P_ during the active stair climbing phase of < 5 W (females: 2.74±0.84 W; males: 3.23±1.25 W).

**Table 2:** Achievement of criteria for V′O_2_max ^a^ (n = 20).

	**Trial 1**		**Trial 2**		**Both trials**	
**Criterion**	**n**	**%**	**n**	**%**	**n**	**%**
Plateau in V′O_2_	12	60	9	45	8	40
HR_peak_ ≥ HR_max_	8	40	7	35	5	25
RER_peak_ ≥ 1.15	16	80	17	85	15	75
**Number of criteria achieved**						
1 criterion achieved	19	95	20	100	19	95
2 criteria achieved	11	55	10	50	10	50
All 3 criteria achieved	7	35	6	30	5	25

V′O_2_, Oxygen uptake; HR, Heart rate; RER, Respiratory exchange ratio

^a^ Values are given in numbers (n) and %

**Fig 5:**
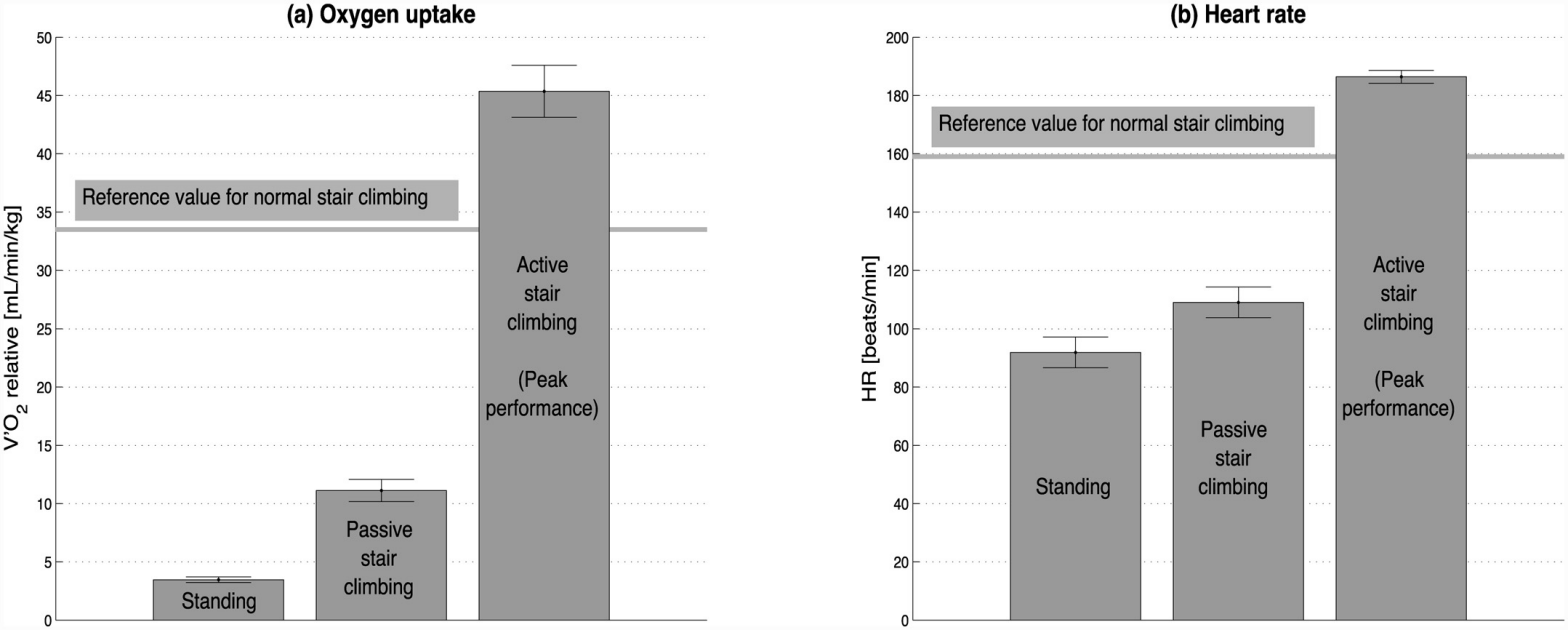
Steady-state values during standing, passive stair climbing, and active stair climbing (peak performance). Steady-state calculations were done using data from the 3^rd^– 4^th^ minutes of a given phase. Values represent the mean ± 95% confidence intervals of the repeated trials. Reference values for normal stair climbing are given for orientation [23].

There was a significant cardiopulmonary response during the transition phases (standing to ‘passive’ stair climbing to ‘active’ stair climbing) (Fig 5). Mean oxygen uptake during standing was 0.29±0.06 L/min and increased to 0.95±0.26 L/min during passive stair climbing (mean oxygen cost of passive stair climbing = 0.66±0.23 L/min, p < 0.001). Mean heart rate during standing was 92±17 beats/min, which increased to 109±17 beats/min (mean heart rate response of passive stair climbing = 17±7 beats/min, p < 0.001). Active stair climbing (mean oxygen uptake 45.36±7.23 mL/min/kg; HR_peak_ 186±7 beats/min) provoked a substantial cardiovascular response that was above the reference values for normal stair climbing (relative V′O_2peak_: 33.5±4.8 mL/min/kg; HR_peak_: 159±15 beats/min) [23].

**Fig 6:**
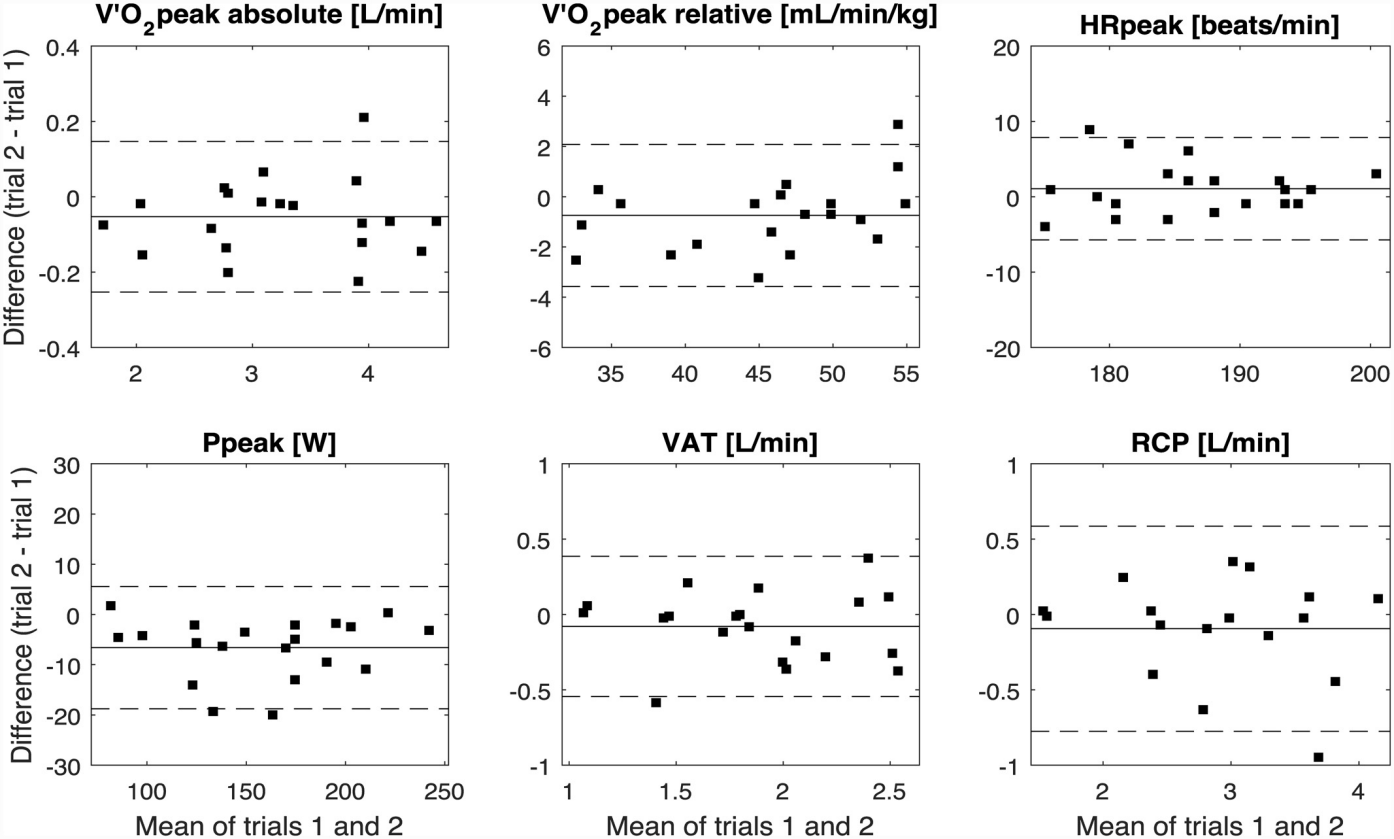
Bland-Altman plots. The difference between trial 1 and trial 2 is plotted against the mean of trial 1 and trial 2 for the primary outcome measures.

The mean values, test-retest reliability and repeatability results of the repeated IETs are shown in Table 3. Practice effects were detected for absolute V′O_2peak_ (p = 0.028), relative V′O_2peak_ (p = 0.029), P_peak_ (p < 0.001), tV′O_2peak_ (p = 0.004), and for RMSE_P_ (p = 0.004). Excellent relative and absolute reliability was found for almost all primary outcome measures (peak values: ICC ≥ 0.890, SEM ≤ 0.60%, MDC ≤ 1.67%). Repeatability for the primary outcomes was good (CoV ≤ 0.12; MD±SD_diff_ for absolute V′O_2peak_ = 0.05±0.10 L/min, relative V′O_2peak_ = 0.74±1.40 mL/min/kg, HR_peak_ = 1.05±3.39 beats/min, P_peak_ = 6.61±6.07 W, VAT = 0.08±0.23 L/min, RCP = 0.09±0.34 L/min). Bland-Altman plots for the primary outcomes of interest show the differences between trials (Fig 6). There were no signs of heteroscedasticity.

**Table 3:** Reliability and repeatability of robot-assisted stair-climbing-based cardiopulmonary exercise testing (n = 20).

	Trial 1	Trial 2										
	Mean±SD	Mean±SD	*p*-value	MD	(LoA)	CoV	ICC	(95%CI)	SEM	SEM%	MDC	MDC%
**Primary outcomes**												
V′O_2peak_ absolute [L/min]	3.23±0.83	3.29±0.83	*0.028	0.05	(0.15–0.25)	0.03	0.991	0.973–0.998	0.01	0.29	0.03	0.81
V′O_2peak_ relative [mL/min/kg]	44.99±7.57	45.73±7.04	*0.029	0.74	(2.06–3.54)	0.03	0.978	0.933–0.992	0.21	0.46	0.58	1.27
HR_peak_ [beats/min]	186.95±7.39	185.90±7.38	0.183	1.05	(5.74–7.84)	0.02	0.890	0.746–0.955	1.13	0.60	3.12	1.67
P_peak_ [W]	155.54±45.19	162.16±45.03	*<0.001	6.61	(5.54–18.76)	0.04	0.981	0.791–0.995	0.84	0.53	2.32	1.46
VAT [L/min]	1.84±0.46	1.92±0.74	0.146	0.08	(0.39–0.54)	0.12	0.869	0.701–0.946	0.08	4.48	0.23	12.42
RCP ^a^ [L/min]	2.84±0.72	2.89±0.82	0.269	0.05	(0.63–0.73)	0.12	0.902	0.757–0.963	0.11	3.72	0.29	10.30
**Secondary outcomes**												
tV′O_2peak_ [min]	8.01±0.95	8.36±1.13	*0.004	0.35	(0.58–1.27)	0.06	0.858	0.519–0.951	0.17	2.14	0.48	5.92
V′_Epeak_ [L/min]	125.91±35.32	127.90±34.18	0.421	1.99	(19.62–23.60)	0.09	0.952	0.886–0.981	2.37	1.87	6.56	5.17
R_fpeak_ [1/min]	51.32±9.59	52.01±8.85	0.555	0.69	(9.60–10.98)	0.10	0.849	0.659–0.937	2.00	3.87	5.54	10.72
RER_peak_	1.22±0.07	1.21±0.06	0.579	0.01	(0.08–0.09)	0.04	0.801	0.564–0.916	0.02	1.59	0.05	4.40
ΔV′O_2_/ΔP_mech_ [mL/min/W]	19.12±2.39	18.62±1.95	0.055	0.50	(1.68–2.68)	0.06	0.858	0.660–0.943	0.41	2.17	1.14	6.03
ΔV′_E_/ΔV′CO_2_	31.02±3.67	31.04±3.69	0.975	0.01	(3.55–3.58)	0.06	0.888	0.738–0.954	0.60	1.92	1.65	5.33
RMSE_P_ [W]	3.36±1.27	2.56±0.59	*0.004	0.80	(1.34–2.93)	0.36	0.323	-0.068–0.649	0.88	29.70	2.44	82.32
ΔV′O_2_ rest-passive [mL/min]	661.36±224.68	655.67±246.03	0.834	5.70	(234.55–245.94)	0.18	0.875	0.712–0.949	42.47	6.45	117.72	17.88
ΔHR rest-passive [beats/min]	17.62±6.71	16.46±8.24	0.273	1.16	(8.05–10.37)	0.27	0.810	0.588–0.920	2.01	11.78	5.56	32.65

SD, Standard deviation; MD, Mean difference; LoA, Limits of agreement; CoV, Coefficients of variation; ICC, Intraclass correlation coefficient; CI, Confidence interval; SEM, Standard error of the measurement; MDC, Minimal detectable change; V′O_2_, Oxygen uptake; HR, Heart rate; P, Work rate (power); VAT, Ventilatory anaerobic threshold; RCP, Respiratory compensation point; t, time; V′_E_, Ventilation rate; Rf, Respiratory rate; RER, Respiratory exchange ratio; V′CO_2_, Carbon dioxide output; RMSE, Root mean square error

^a^ n = 17

* p < 0.05

## Discussion

The aim of this study was to evaluate the feasibility, reliability, and repeatability of augmented RASC-based CPET in able-bodied subjects, with a view towards future research and applications in neurologically impaired populations. The driving questions were: (1) does the approach provide substantial cardiopulmonary responses appropriate for CPET; (2) is the feedback-control approach technically implementable; and (3) is the approach able to reliably determine V′O_2_max, sub-maximal ventilatory thresholds, and other key outcomes?

The results demonstrate cardiopulmonary responsiveness and successful technical implementation of RASC-based CPET. Predicted values of V′O_2max_ and HR_max_ were achieved or surpassed (mean V′O_2peak_ = 106±9% of predicted V′O_2max_; mean HR_peak_ = 99±3% of predicted HR_max_). Although reference data on treadmill-based CPET in a comparable sample [24] showed slightly higher values (mean V′O_2peak_ absolute: 3.55 L/min vs. 3.26 L/min here; mean V′O_2peak_ relative: 48.70 mL/min/kg vs. 45.36 mL/min/kg; mean HR_peak_: 195 beats/min vs. 186 beats/min), RASC-based CPET yielded higher cardiopulmonary responses compared to published data for leg cycle ergometry (mean V′O_2peak_ absolute: 2.81±0.19 L/min; mean HR_peak_: 179±8 beats/min) [25] and total body recumbent stepper exercise (mean V′O_2peak_ absolute: 3.13±0.80 L/min; mean HR_peak_: 182±13 beats/min) [26]. Furthermore, RASC-based CPET has been shown to be able to fulfil criteria for V′O_2max_, an important aspect during CPET. All subjects achieved at least 1 criterion during the 2 trials (trial 1: 95%; trial 2: 100%). This distribution is comparable to published data on treadmill-based CPET (plateau in oxygen uptake: 20% vs. 40% here; HR_peak_ ≥ predicted HR_max_: 26% vs. 25%; RER_peak_ ≥1.15: 69% vs. 75%) [27]. Despite criticism of the criteria used to establish whether a true V′O_2max_ has been attained [28], the results presented here clearly reveal the comparability of RASC-based CPET to conventional exercise testing approaches.

The successful identification of the VAT (100% of the subjects and trials) and the RCP (95% of the subjects; 90% of the trials) provides an additional means for estimation of cardiovascular fitness and exercise prescription. This is an important finding for future applications in populations who cannot undergo maximal CPET due to their methodological ineligibility or high-risk profile for adverse events. The VAT as a percentage of V′O_2_peak (58±6%) and RCP (86±6%) found in this study were in the higher range (reference values: VAT: 40–60%; RCP 60–90% [29]), which underlines the good physical fitness of the subjects included.

A critical aspect during CPET is the definition of the target work rate slope in order to reach peak cardiovascular performance (tV′O_2peak_) between 8 and 12 min [3, 4]. The present experimental protocol aimed to define the slope by predicting P_max_ at baseline using maximal voluntary effort testing (the subjects had to follow a steep slope until they reached their power limit). The findings indicate that this methodology overestimates P_max_ (females: p < 0.001, males: p = 0.004). Consequently, the steeper target work rate slope, as a result of the overestimation, affects tV′O_2peak_, which actually was in the lower range (females: 8.12±1.22 min, males: 8.26±0.81 min). Similarly to established protocols using leg cycle ergometry and treadmill exercise, there is a challenge of predicting a subject’s P_max_ to estimate the optimal target work rate level. Based on the findings of this study, 85% (females: 84%, males: 87%) of predicted P_max_ using maximal voluntary effort testing could be used as a reference to define the target work rate slope for RASC-based CPET. However, further analyses in large target populations are needed to confirm this finding and establish appropriate reference data.

The fact that all included subjects achieved an RMSE_P_ below 5 W clearly shows the successful technical implementation of the feedback-control system. This is an important finding since end-effector-based feedback-control systems can simulate repetitive task-specific exercise, which is of high interest for rehabilitation purposes. Whether this approach is feasible in severely impaired individuals should be a focus of future studies. A major challenge will be the control of optimal muscle activation patterns during end-effector based stair climbing in populations with neurological impairments of the lower extremities. Several factors such as the application of body weight support, the level of neuromuscular control, and the cognitive state of the subject will be challenges for clinical implementation.

The findings presented here suggest that RASC is a feasible approach to assess peak cardiopulmonary performance and ventilatory thresholds, and could, therefore, serve as an alternative that promotes repetitive task-specific exercise for training and testing of cardiovascular fitness in populations with severe neurological impairments. The novel approach achieved higher peak cardiopulmonary performance values than conventional approaches, which is an important finding regarding the evaluation of true maximal exercise capacity. Furthermore, the results demonstrate that conventional end-effector-based robot-assisted stair climbing is not passive, which could be of high importance for cardiovascular exercise prescription in severely impaired populations who cannot achieve required target work rate levels.

Regarding reliability and repeatability, high precision has previously been demonstrated for cardiopulmonary performance measures [4]. The present study demonstrates similar findings for this new exercise modality with excellent relative and absolute reliability for the most important peak performance parameters (ICC ≥ 0.890; SEM ≤ 0.60%; MDC ≤ 1.67%). RASC-based CPET has high repeatability as determined by the MD and Bland-Altman’s LoA (Table 3), and CoV were comparable to previously published data in both apparently healthy and chronic disease cohorts (V′O_2peak_: 0.03–0.09 vs. 0.03 here; HR_peak_: 0.01–0.09 vs. 0.02; VAT: 0.09–0.13 vs. 0.12) [4]. However, significant differences between trial 1 and trial 2 were found for absolute and relative V′O_2peak_, P_peak_, tV′O_2peak_, and RMSE_P_, which means that subjects consistently performed better in the second trial. This finding can be explained by the practice effect, i.e. motor learning during the experimental procedure leads to more efficient performance over time, which can be seen in the decrease in RMSE_P_ and the increase in V′O_2peak_, P_peak_, and tV′O_2peak_ from trial 1 to trial 2. The subjects learned how to generate required work rate levels to perform longer over time (increase in tV′O_2peak_) and in higher intensity ranges, which led to higher V′O_2peak_ values in trial 2. This result emphasises the importance of the familiarisation procedure with the augmented RASC approach, especially when applied in populations with motor impairments of the lower limbs. The findings on reliability and repeatability suggest that RASC-based CPET is a precise supplemental concept for assessment of cardiovascular fitness, which might have potential for functional CPET in neurological rehabilitation settings.

Overall, the results demonstrate potential for augmented RASC to be used for exercise testing and prescription in populations with neurological impairments who benefit from repetitive task-specific training, as the approach has been shown to be feasible, reliable, and repeatable in able-bodied subjects. Future research should focus on the clinical implementation.

This study has some limitations. (1) The small sample size of the study may render the results underpowered, since at least 50 subjects are generally seen as adequate for the assessment of the agreement parameter [30]. (2) The comparison of V′O_2peak_ and V′O_2max_ were based on predicted values. A comparison of RASC-based CPET with a maximal stair climbing exercise tests in the field using mobile cardiopulmonary monitoring systems might provide a deeper insight into the validity of this novel concept. (3) Subjects were instructed to hold the handrails for safety reasons, which might have led to additional muscular activity of the upper extremities. This could have led to higher peak performance values when compared to leg cycle ergometry or treadmill exercise, since the amount of muscle mass involved has an influence on V′O_2max_ [31]. (4) The present study protocol strictly controlled time of day for CPET, and the tests were performed within 48 h or 72 h. This time difference might have affected the recovery phase of the subjects, thus influencing the results. Furthermore, some of the day-to-day variability (normally ±3% [4]) between the tests may be related to uncontrolled sources of variance, like food and beverage intake, but also to daily differences in subjective symptoms like fatigue.

## Conclusions

This study established feasibility, together with reliability and repeatability, for augmented RASC-based CPET in able-bodied individuals. The end-effector-based approach demonstrated comparable or higher peak cardiopulmonary performance variables relative to predicted values, it achieved the criteria for V′O_2max_, and it allowed determination of sub-maximal ventilatory thresholds. The reliability and repeatability were found to be high. There is potential for augmented RASC to be used for exercise testing and prescription in populations with neurological impairments who would benefit from repetitive task-specific training.

## Supporting Information

S1 Dataset Outcome measures during repeated robot-assisted stair-climbing-based cardiopulmonary exercise testing. (XLSX)
